# Clinical spectrum of pseudoexfoliation syndrome—An electronic records audit

**DOI:** 10.1371/journal.pone.0185373

**Published:** 2017-10-27

**Authors:** Aparna Rao, Debananda Padhy, Prity Sahay, Amiya Pradhan, Sarada Sarangi, Gopinath Das, Niranjan Raj

**Affiliations:** Glaucoma Service, LV Prasad Eye Institute, Patia, Bhubaneswar, Odisha, India; Universita degli Studi di Firenze, ITALY

## Abstract

**Purpose:**

To evaluate different clinical variants of pseudoexfoliation syndrome and their risk of developing ocular hypertension (OHT) or glaucoma (PXG)

**Design:**

Cross sectional hospital based study.

**Setting:**

All patients seen at glaucoma services of a tertiary eye care center in east India.

**Methods:**

Electronic medical records search of hospital database including consecutive new and old cases seen during April 2013 to March 2015 was done to retrieve case sensitive words including pseudoexfoliation, PXF, PEX, PXG and pseudoexfoliative glaucoma over any part of the clinical electronic sheet of the patient. All demographic and clinical details including laterality, the pattern of deposits, need for medicines and disc damage at presentation was compared in eyes with radial pigmentary, classical or combined forms of PXF phenotypes.

**Results:**

Of 110313 PXF patients seen during the period of 2013–2015, a total of 2297 eyes of 1150 PXF patients were identified including 525 unilateral PXF (meaning a total of 1775 PXF eyes with 625 patients having bilateral disease, n = 1250 eyes, other clinically normal eye, n = 522) at presentation. Of 525 unilateral PXF eyes, 105 had OHT and 131 had glaucoma while bilateral cases had more >50% (675 eyes of 1250 eyes) with glaucoma. Glaucoma with significant changes in IOP with or without disc damage was seen in 32% of pigmentary and 39% of classical PXF forms with eyes with combined forms of PXF having around 50% with glaucoma at presentation compared to other forms, p<0.001.

**Conclusion:**

Different phenotypic variants of PXF in this Indian cohort was associated with 30–50% risk of OHT or glaucoma respectively. Adequate care is required while examining the pattern of PXF in each case to prognosticate each patient/eye.

## Introduction

Pseudoexfoliation (PXF) and pseudoexfoliation glaucoma (PXG) is an age related fibrillopathy with varied prevalence worldwide.[1–5]The ocular and systemic associations of this disease are well described in literature mandating its identification as a unique entity among different types of glaucoma.[2–4] The most unique and characteristic ocular feature which sets the disease apart is the presence of dandruff like pseudoexfoliative material (XFM) over different parts of the including the lens, pupil or cornea. The pathogenesis of the disease still remains a mystery with several factors including genetic, environmental or diet factors being implicated in XFM formation.[1,3]While literature on the incidence of pseudoexfoliation glaucoma (PXG) and ocular hypertension (OHT) due to the disease varies across different parts of the globe, details on the vast clinical spectrum of the disease and the frequency of blindness due to PXF at presentation are scarce in literature with most studies focussing on genetic aetiology, pathogenesis or prevalence of the disease.[5–7] The cause for glaucoma in PXF is unclear and assumed to be due to various reasons including mechanical blockage of the trabecular meshwork (TM) by XFM and ischemic or molecular insults which cause irreversible tissue damage.[1,2]It is now known that the extent of XFM, angle pigmentation or disease severity of presentation has no correlation with risk of OHT or glaucoma. While genetic and environmental conditions may predispose to XFM formation or glaucoma, absence of concurrence of these risk factors across ethnic populations suggest clinical or local eye specific risk factors which may predispose to PXG or OHT.[1,8–12] While features of early stages or unmanifest stage of the disease is described which also includes radial pigments over the lens capsule,[2,3] specific clinical phenotypes which may signify a risk for developing OHT or future glaucoma in such phenotypic variants is unknown or not explored. This information would identify the risk of raised intraocular pressure or glaucoma in high-risk eyes allowing timely intervention and closer follow up. We had earlier described varied phenotypes of this entity seen in our region with varied clinical features suggestive of early unmanifest disease. We identified several clinical features which may signify the early onset of PXF without the evident classical dandruff like deposits and described features that were associated with the presence of glaucoma. We also characterized three stages of the disease which needed further evaluation for assessing the differences in risk for progression to ocular hypertension or glaucoma in each stage of the disease. Collecting this information to identify the specific risk of OHT and glaucoma in each stage of PXF would allow formulation of specific guidelines for managing this disease more appropriately at each stage. We now present the demographic and clinical spectrum of patients with pseudoexfoliation seen at a tertiary center and also evaluate the various phenotypic variants and the glaucoma profile in each stage of the disease described earlier.

## Methods

This was a hospital electronic medical records audit of hospital record database at a tertiary eye center in East India of consecutive new and fresh cases seen during April 2013 to March 2015. The institutional electronic medical record (EMR) includes all data regarding the patient demographic and clinical details, surgeries, investigations, medications, and other details all uploaded and stored onto a central server which can be later retrieved. The EMR database retrieved all cases by identifying case sensitive words including pseudoexfoliation, PXF, PEX, PXG and pseudoexfoliative glaucoma over any part (part of the optometrist, perimetrist, ophthalmologist or diagnostic investigation parts of the clinical sheets) of the clinical electronic sheet of the patient. This study was approved by the institutional review board of LV Prasad Eye Institute, Bhubaneswar and adhered to the tenets of Declaration of Helsinki. Details retrieved included demographic variables and slit lamp evaluation with slit lamp photographs, best corrected Snellen visual acuity, intraocular pressure (IOP) by Goldman applanation tonometry, +90D fundus biomicroscopy and Humphrey visual fields (Carl Zeiss Inc, Dublin, CA, USA, 24–2 SITA standard program). Details of referrals from outside, the pattern of deposits, diagnosis, ocular associations at presentation and surgical course were retrieved from the EMR database.Pseudoexfoliation was diagnosed in eyes with evident classical dandruff pattern of deposits on the pupil, lens or other ocular structures, radial pigment over the lens surface after dilated slit lamp evaluation. The PXF forms were classified into three clinical phenotypes based on the clinical features described in our earlier study.[9] Briefly it is as follows- a) Radial pigmentary, RP, form (earlier described pre-granular form)–The clinical features pointing in these eyes would be radial pigments with or without pupillary ruff atrophy. b) RP with classical with early coalescence of pigmentary deposits into the classical form of peripheral exfoliation deposits or combined forms of classical with true exfoliation.c) Classical PXF with dandruff like deposits in the peripheral and /or central area but not restricted to pupillary ruff, lens surface or cornea.Eyes with clinically evident PXF and normal IOP/visual field and optic nerve were classified as those with pseudoexfoliation syndrome. Eyes with PXF with raised IOP >21mm Hg with normal optic nerve and visual field were diagnosed as PXF with OHT after exclusion of other types of glaucoma. Glaucoma (PXG) was defined as those with glaucomatous optic neuropathy evidenced by cupping, rim thinning, notch or retinal nerve fibre layer defects with corresponding visual field defects. Visual field defects were classified as glaucomatous if glaucoma hemifield test outside normal limits or pattern standard deviation with probability <5%, which were reproducible over three baseline fields. Eyes with prior medications, history of filtering surgeries, laser or other interventions for controlling IOP were included in the presence of XFM in the eye. Both unilateral and bilateral disease with above features were included in the study. The contralateral eye with no evident XFM in the eye and normal IOP, optic nerve and visual field was considered as the clinically normal eye. Patients with any other autoimmune or neurodegenerative disorder and diabetes mellitus were excluded.

### Statistics

All analysis was done using Stata Corp (USA, Version 10) with alpha error set at p<0.05. Since both unilateral and bilateral cases were included, generalized estimation equation was used for assessing the association of phenotypic features with OHT/glaucoma which accounts for correlation between two eyes of the same patient. Continuous variables are represented as mean (+SD) or Media (range) while frequency of features are represented as numbers (n,%).

## Results

**Fig 1:**
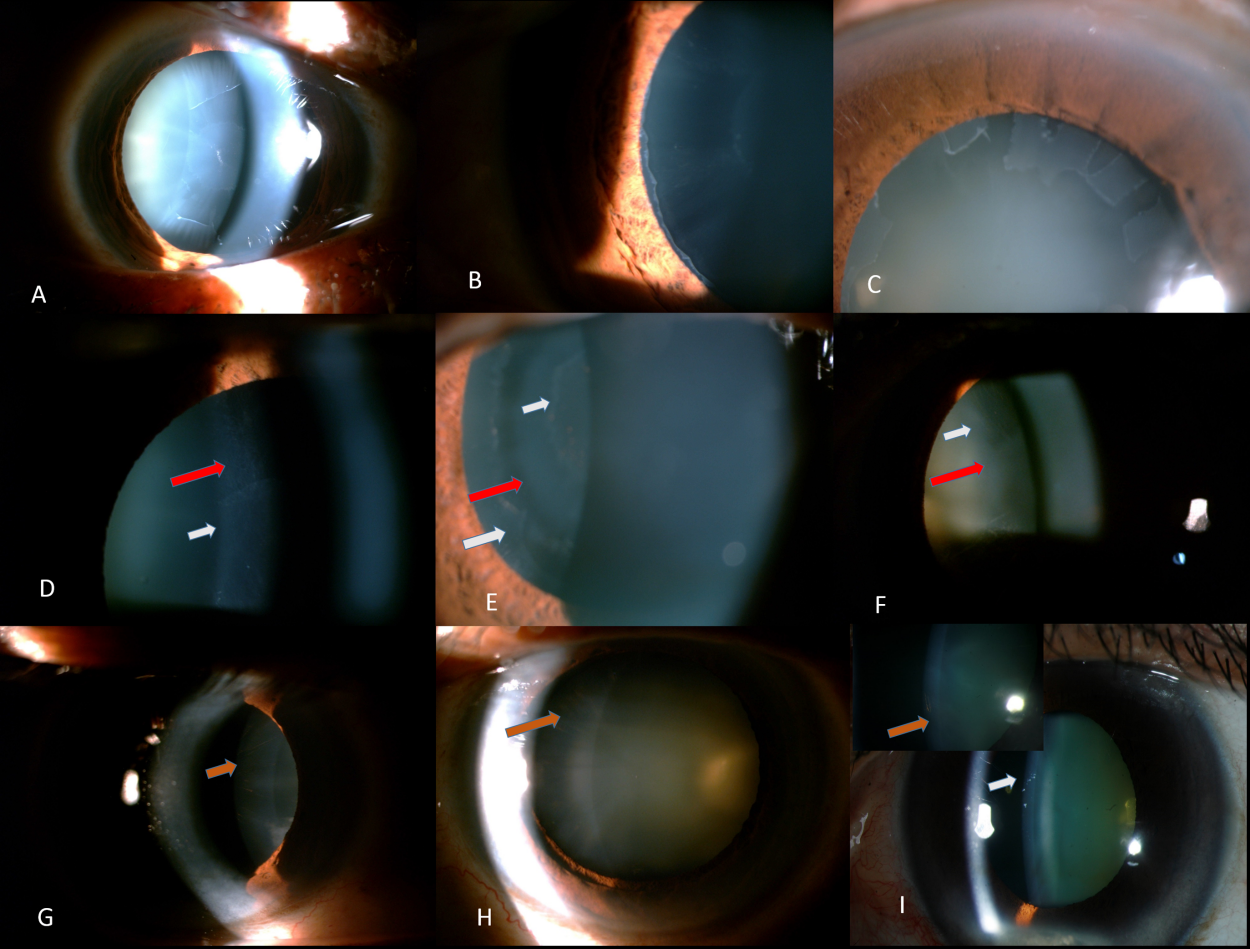
Representative cases of different forms of pseudoexfoliation. A-shows the classical three ring sign (white arrows) while B shows an eye with prominent peripheral ring and clear space and subtle central ring and C shows another variant with rectangular pattern of deposits conforming to the iris pattern shown above the deposits. D-F shows different variant forms of pseudoexfoliation deposits which are diffuse or in a classical ring pattern or combined with other forms like true exfoliation (red arrow). G-I shows form of pigmentary pseudoexfoliation showing radial pattern of deposits in the periphery (G) in isolation or combined with classical deposits (H and I).

**Fig 2:**
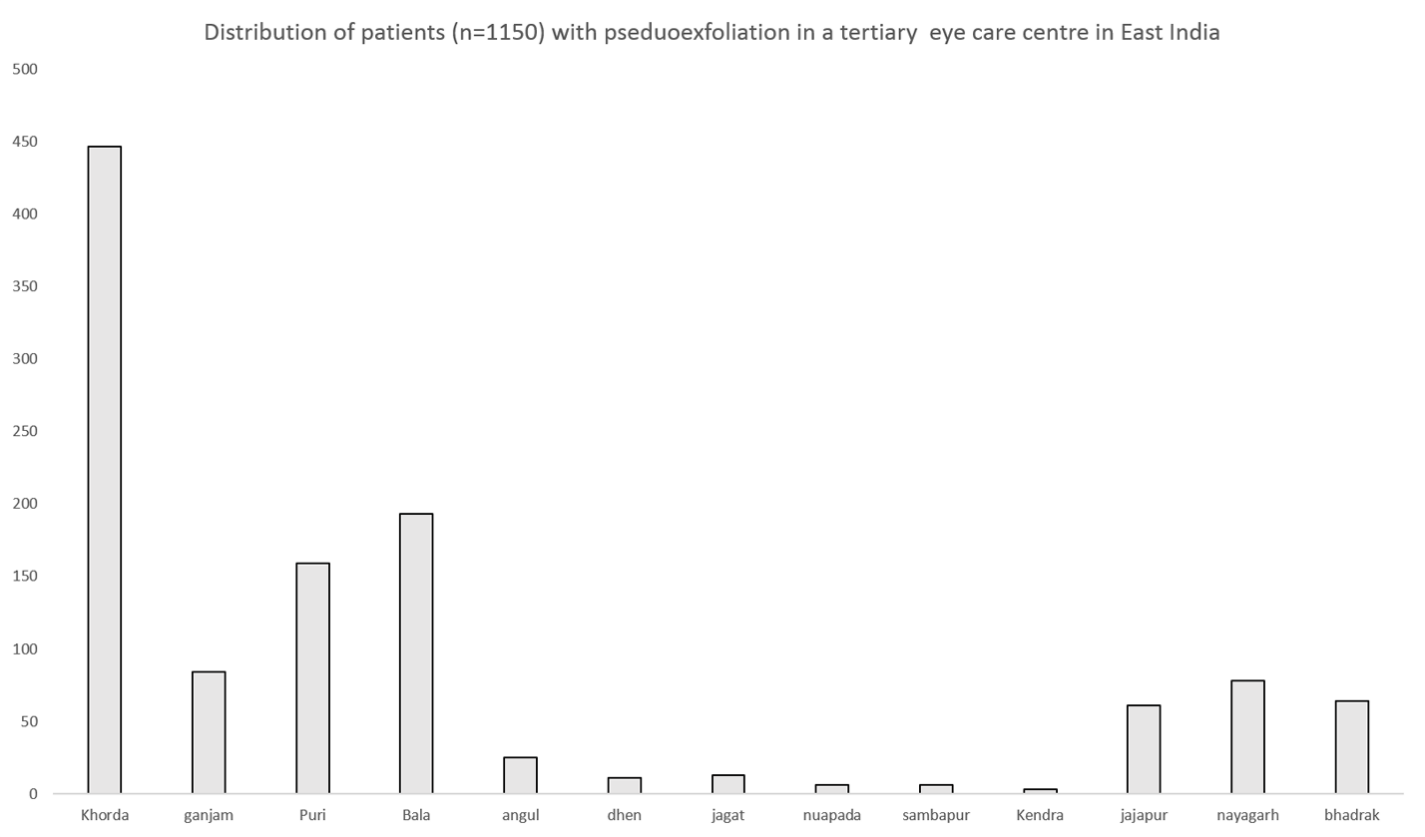
Shows the geographical distribution of patients with pseudoexfoliation from different districts of Odisha, East India.

Of 266867 ophthalmic cases and 110313 new cases seen during the period of 2013–2015, a total of 2297 eyes of 1150 new patients (3 one eyed patients at presentation) with pseudoexfoliation were identified from the hospital EMR database. This included 525 unilateral PXF cases which also included 3 one eyed patients at presentation. This meant a total of 1775 PXF eyes with 625 patients having bilateral disease (1250 eyes) at presentation while 525 had unilateral disease (other clinically normal eye, n = 522) at presentation, **Table 1**. **Fig 1** shows some representative cases of each form of PXF seen in this cohort.Looking at areas of origin, 233 cases were from Khorda district of Odisha, **Fig 2**. This included 866 males with only 4.6% (n = 106) cases being referred from other practitioners. Of those referred, only 9 cases carried the diagnosis of pseudoexfoliation while the rest were referred as primary open angle glaucoma and 3 were referred for retinal vein occlusion. While 75% came for routine eye check-up or for presbyopic glasses, rest sought eye care services for cataract or other problems like glaucoma. Ocular associations in these eyes included RVO (n = 12), microbial keratitis (n = 16), retinal detachment (n = 11) and retinitis pigmentosa (n = 3), 1 myasthenia gravis, 1 Dry ARMD, 3 NLDO. Of 1775 eyes, 1124 eyes had vision <20/200 at presentation with 28 eyes having absolute glaucoma with majority having poor vision due to visually significant cataract, **Supplemental S1 Table**. A total of 38 eyes with glaucoma had poor vision due to end stage disease or ocular associations like corneal scarring.

**Table 1:** Baseline characteristics of patients with pseudoexfoliation in a tertiary eye care centre.

Variables	Frequency
Unilateral: Bilateral: Clinically normal (N, eyes)	525:1250:522
Male: Female	284:866
Referred/primary	106:2191
PXF syndrome/glaucoma (n, eyes)	1094 syndrome:Glaucoma:447OHT-234clinically normal eye of unilateral cases: 522
Type of glaucoma (%)	PACG-13.7%NTG-0.8%Lens induced—3.7%POAG-81.7%
Pattern of exfoliation depositsN eyes, %	Classical-1024Pigmentary -43 (3.9%)Combined-13True-14
Lens subluxation (n, eyes)	46
Lens grade (N, eyes)	NS1- 466NS2-653ns3- 248>ns4-101ACIP-13PCIOL-265

OHT-Ocular hypertension; PXF-Pseudoexfoliation; PACG-Primary angle closure; POAG-primary open angle glaucoma; NTG-Normal tension glaucoma

### Unilateral/bilateral presentation

Of 1775 eyes, 525 eyes had unilateral disease which included 3 patients who were one eyed at presentation having lost the other eye owing to trauma or microbial keratitis. Bilateral eyes tended to present at marginally later age and with glaucoma (66% compared to 35% of unilateral presenting with glaucoma), **Tables 2 and 3**. Unilateral cases had an almost equal proportion of eyes with OHT (20%, 105 of 525 eyes) and glaucoma (25%, 131 of 525 eyes) while bilateral cases had more >50% (675 of 1250 eyes) with glaucoma.

**Table 2:** Clinical profile of patients with different stages of pseudoexfoliation in a tertiary eye care centre.

Variables	Pseudoexfoliation OHTN = 234	PseudoxfoliationGlaucomaN = 447	Pseudoexfoliation SyndromeN = 1094	Clinically normal eyeN = 522	P value
Age (years)	69±9.8	71±9.7	70±8.9	73±9.6	0.6
Baseline IOP (mm Hg)	19±8.1	25±13.1	16±5.5	14±3.9	<0.001
Male: Female	185:49	367:80	829:265	417:105	0.7
Baseline MD (dB)	-12-2±9.1	-20±9.2	-10±7.6	-8±8.1	<0.001
Baseline PSD (dB)	10±3.2	6±3.1	4±2.9	5±3.3	<0.001
VFI (%)	66±33.2	33±29.2	72±30.6	79±19.7	<0.001
Medicines at presentation (n, eyes)	0 meds-831 meds-582meds-643 meds-244meds-5	0 meds-1691meds-542meds-1243meds-714meds-29	NA	NA	<0.001
Finding or diagnosis (N)	Both-81Finding-39Diagnosis-114	Both-163Finding-73Diagnosis-211	Both 712Finding 343Diagnosis 39	NA	<0.001

OHT-Ocular hypertension; PXF-Pseudoexfoliation;IOP-Intraocular pressure; MD-Mean deviation; PSD-Pattern standard deviation; VFI-Visual field index

**Table 3:** Clinical characteristics in 1775 eyes with pseudoexfoliation in an electronic medical audit and their comparison with clinically normal eyes of patients with unilateral exfoliation (n = 522).

Variables	UnilateralN = 525 eyes			BilateralN = 1250 eyes			Normal eyesN = 522
Age (years)	69±9.8			70±9.2			69±9.9
Baseline IOP (mm Hg)	19±9.1			27±18.4			16±8.2
Lens subluxation (n, eyes)	7			39			0
Stages of pseudoexfoliation%, n, eyes	ES	OHT	Glaucoma	ES	OHT	Glaucoma	
	55% 289	20% 105	25% 131	34% 425	12% 150	54%675	NA
Diagnosis missing with only finding (n, eyes)	331			124			NA
Location (N, eyes)	Both 226Pupil 292Lens7			Both 221Pupil 1021Lens 8			NA

OHT-Ocular hypertension; PXf-Pseudoexfoliation;IOP-Intraocular pressure, ES-Exfoliation syndrome with normal disc and IOP

### Distribution of phenotypic pattern of exfoliation deposits and OHT/glaucoma in each phenotype

Of 1775 eyes, the classical pattern was noted in 1693 eyes while others had pigmentary PXF (n = 69), combined forms of PXF, **Tables 3 and 4**. Of all patients, 16 had true exfoliation combined with classical peripheral PXF ring with central disc plaque and early lens capsule splitting, **Fig** 1. Glaucoma with significant changes in IOP with or without disc damage was seen in >1/3^rd^ of both pigmentary and classical forms of PXF with eyes while combined forms of PXF had more bilateral disease forms with around 50% having glaucoma at presentation, **Table 4**. Of those with pigmentary PXF, 16 eyes of 9 patients were on 1 or >1 medicines for IOP control while those with combined forms (n = 6) required >2 glaucoma medicines for IOP control.

**Table 4:** Clinical phenotypes and forms of pseudoexfoliation in an electronic medical audit at a tertiary eye care centre.

Variables	PigmentaryN = 69			ClassicalN = 1693			CombinedN = 13			P
Age(years)	68±9.3			70±9.5			71±10.4			0.6
Baseline IOP (mm Hg)	17±7.2			28±19.4			27±10.5			<0.001
Lens subluxation (n, eyes)	0			46			0			NA
Males (N, %)	3753.6%			52130.7%			1076.9%			<0.001
Stages of pseudoexfoliation	ES	OHT	Glaucoma	ES	OHT	Glaucoma	ES	OHT	Glaucoma	
%, n	68% 47	12% 8	20% 14	61% 1035	9% 152	30% 506	38.4% 5	20.6% 2	41% 6	<0.001
Unilateral eyesN, %	68.6%			51730.5%			27.6%			<0.001
VFI (%)Mean± SD	70±34.5			63±33.7			88±9.1			<0.001

OHT-Ocular hypertension; PXF-Pseudoexfoliation;IOP-Intraocular pressure; MD-Mean deviation; PSD-Pattern standard deviation; VFI-Visual field index, ES-Exfoliation syndrome with normal disc and IOP

### Clinical attributes of exfoliation glaucoma

Of 1775 PXF eyes, while 681 eyes had glaucoma (n = 447, 25.1%) or ocular hypertension (n = 234, 13.1%) with 3 eyes being atrophic/phthisis bulbi at presentation, **Tables 3 and 4**. Glaucoma type was closed angle in 13% eyes with lens induced glaucoma seen in 3.7% eyes and 81% resenting as open angle glaucoma. A total of 28 eyes presented with absolute glaucoma with 14 eyes being blind due to end stage neovascular glaucoma secondary to previous vein occlusive disease. Among eyes with OHT and glaucoma, 93 eyes in OHT group and 225 eyes required>2 anti-glaucoma meds (including combination of beta blockers, prostaglandins, alpha-agonists and topical dorzolamide)for IOP control at presentation with 5 and 29 eyes requiring 4 or >4 medicines in each group, respectively. Of 39 eyes with OHT where the diagnosis was missing (Table 2), 28 eyes received one medication for IOP spike noted in office visit before or after cataract surgery without a detailed glaucoma evaluation ordered at any visit. Few eyes in the glaucoma group (n = 169) did not receive any medicine owing to either very poor visual prognosis in the latter group (n = 128 with 28 absolute glaucoma eyes) or status post trabeculectomy elsewhere (n = 8) with the rest being lost to follow up after the initial visit where glaucoma evaluation was advised. Of those with OHT, 83 eyes did not receive medicines because of either transient spikes post cataract surgery (n = 69) or were angle closure which was relieved after LPI (n = 14).

### Surgical indications, course, and outcomes in exfoliation

The pupillary dilatation status was ≤4mm in 312 eyes while it was not mentioned in 493 eyes. Of 1775 eyes, 349 eyes had nuclear grade >3 with posterior dislocation of the lens in 8 eyes. All non-dilating pupils were managed successfully during surgery with one eye experiencing iridodialysis in 3 quadrants during nucleus delivery. A total of 27 eyes had leucomatous or macular corneal scarring involving central visual axis causing visual impairment. A total of 13 eyes had bullous keratopathy (Pseudophakic, n = 8), Aphakic, n = 5) at presentation due to prior surgery. Of 1775 eyes with glaucoma, 385 underwent small incision cataract surgery with intraocular lens (SICS+IOL), 3 underwent SICS and trabeculectomy, 135 underwent phacoemulsification with IOL, 80 SICS with anterior vitrectomy with trabeculectomy, 27 phacoemulsification with trabeculectomy, 6 trabeculectomy with Mitomycin-C while pars plana lensectomy was done in 8 eyes with posterior dislocated lenses. Bleb repair status post trabeculectomy done outside was required in 3 eyes with three requiring 1–2 medicines after repair. A total of 76 eyes had intraoperative zonular dialysis or PC rents with IOL being placed successfully in 85% of cases. Cases where the diagnosis was missing (n = 58) had more incidence of intraoperative complications than those with documented diagnosis, (n = 18). The best corrected final visual acuity improved by >2 Snellen lines of visual acuity in all except 3 eyes. One eye had cystoid macular edema which required intravitreal steroid injection which led to the development of steroid glaucoma and uncontrolled IOP at 1.8 years mandating surgery.

## Discussion

This EMR based audit found increased OHT/glaucoma in eyes having combined forms of the disease at presentation. Bilateral disease is known to present at a later age and have greater severity at presentation due to chronic damage compared to unilateral disease.[**1,2,10,12,13–15**] While bilateral cases were more common in classical form of PXF, this clearly did not explain the higher prevalence of OHT/glaucoma in combined forms suggesting other mechanisms of TM and glaucoma damage in different phenotypic forms of PXF. Earlier epidemiological studies have reported different rates of OHT and glaucoma in PXF or prevalence of PXF which varies across studies due to differences in inclusion criteria and difference in baseline disease prevalence across ethnic populations.[**5,6,10,13,14,16–26**]. The Blue Mountains study found 52% of unilateral and 48% of bilateral PXF cases in their study.^20^ Several studies have also reported high conversion rates of unilateral to bilateral cases with the Reykjavik study reporting a 70% conversion over 12 years.[**16**] In the Thessaloniki Eye Study, the proportion with glaucoma among pseudoexfoliative participants (15.2%) was higher than that for glaucoma among non-pseudoexfoliation participants (4.7%). In the Reykjavik Eye Study, the overall 12-year incidence for either eye increased from 6.5% in participants aged 50–59 years at baseline to 10.6% in those that were 70–79 years at baseline.[**21**] The Blue mountain study reported glaucoma in 14% and OHT in 9% eyes in an Australian population while >50% had glaucoma in a study on Turkish population.[**5,20**] Another survey of 1184 Australians revealed a PXF prevalence of 4.7% while none had glaucoma or OHT.[**25**] Our study found different risk of OHT/glaucoma in eyes with different phenotypic patterns of PXF apart from differences in baseline parameters like IOP and laterality. Earlier studies have focussed on clinical features and various facets of the classical form recognized in late stages possibly because of lack of importance given to the varied phenotypic pattern of the deposits or their significance or even lack of dilated examination to identify different patterns of PXF which are rarely studied or reported. Earlier studies only report the overall prevalence of OHT and glaucoma in PXF in general while the risk of developing glaucoma in different phenotypic patterns have not been elucidated. The pre-clinical stage described in literature includes eyes with radial pigments which therefore may be considered to have normal IOP and optic nerve.[2,3] We found 69 eyes with pigmentary forms of PXF in this cohort with 1/3^rd^ of eyes with pigmentary and classical forms associated with raised IOP or glaucoma while >1/2 of eyes with combined form of PXF having glaucoma at presentation. It may be possible that combined forms of glaucoma would have mechanical blockage of the TM with XFM apart from release of pigments suggesting different molecular insults in different PXF forms. While pigment release is also postulated in classical forms of PXF, such mechanisms may be more predominant in eyes with pigmentary/combined forms making documentation of the phenotypic variants important in clinical practice. The prevalence of PXF varies widely across the globe ranging from 0% in Eskimos, 1–2% in the United States, 5% in Turkish population to >25% in Scandinavian countries and Navajo Indians and between males and females.[**1,5,6,13,14,27–29]** Some postulated reasons included differences in environment, diet and lifestyle, differences in hours of outdoor exposure and distance from equator apart from basic difference in genetic susceptibility study design and lack of using slit lamp under dilated examination in many studies. In the Reykjavik Eye Study, prevalence increased from 2.5% in those aged 50–59 years to 40.6% in those aged≥ 80 years. A south Indian based population survey evaluating patients at baseline and at 6 years found an incidence of 2% exfoliation, glaucoma in 1% and 2% OHT incidence.[**17,18] We found a prevalence of 11.5% in a tertiary eye care center in East India with a prevalence of 25% of exfoliation glaucoma and 13% with OHT in this hospital based survey which was higher in combined forms of PXF than even classical form of the disease. This study was an EMR based audit of a single center with its inherent biases due to possible faulty entry affecting data retrieval which cannot be directly extrapolated to population prevalence.** Our study found a high proportion of cases where diagnosis was written merely as a finding on pupil or the lens rather than a diagnosis. One study reported that this finding may be missed even in enucleated eyes where, among 323 non-selected eyes enucleated following painful amaurosis, pseudoexfoliation syndrome was found in 3.4%.[30] This is further compounded by the frequent lack of full dilatation which may be a potential cause for missed diagnosis. Transmission electron microscopy of unselected non-serial sections revealed a precapsular layer in 18 of 28 eyes consisting of typical PEX fibers or microfibrils, which indicated early stages of PXF. Lack of awareness of the earlier signs of PXF and clinical features signifying possible PXF is another cause for missed diagnosis which therefore mandates careful examination for PXF pattern under dilated examination. This was a hospital based cross sectional study from one part of East India which may, therefore, have a selection bias and therefore cannot be generalized. It is possible that some cases may have been missed due to faulty entry or alternative short forms for describing the disease used by some practitioners. Long term studies in the future would nevertheless confirm the differences in baseline risk of OHT/glaucoma in different clinical forms of PXF mandating clear and precise documentation of the phenotypic variant important for assessing future risks in eyes with PXF.

## Supporting information

S1 Table Visual acuity in eyes with pseudoexfoliation syndrome, ocular hypertension and exfoliation glaucoma. (PDF)

## Abbreviations

PXF: Pseudoexfoliation syndrome
PXG: Pseudoexfoliation glaucoma
IOP: intraocular pressure
XFM: exfoliative material
EMR: electronic medical record
TM: trabecular meshwork
OHT: Ocular hypertension

## References

[1] Aboobakar IF, Johnson WM, Stamer WD, Hauser MA, Allingham RR. Major review: Exfoliation syndrome; advances in disease genetics, molecular biology, and epidemiology.Exp Eye Res. 2016; 11;154:88–103 10.1016/j.exer.2016.11.011 27845061

[2] Vesti E, Kivelä T. Exfoliation syndrome and exfoliation glaucoma. *Prog Retinal Eye Res* 2000;19:345–68. 10.1016/s1350-9462(99)00019-1 10749381

[3] Layden WE, Shaffer RN. Exfoliation syndrome. *Am J Ophthalmol* 1974;78: 835–41. 4421811

[4] Naumann GO, Schlotzer-Schrehardt U, Kuchle M. Pseudoexfoliation syndrome for the comprehensive ophthalmologist. Intraocular and systemic manifestations. Ophthalmology 1998;105: 951–968. doi: 10.1016/S0161-6420(98)96020-1 9627642 10.1016/S0161-6420(98)96020-1

[5] Gunes A, Yasar C, Tok L, Tok O. Prevalence of Pseudoexfoliation Syndrome in Turkish Patients with Senile Cataract.SeminOphthalmol. 2016; 21:1–5. 10.3109/08820538.2015.1068344 26795697

[6] Ostenfeld-Akerblom A. Pseudoexfoliation in Eskimos (Inuit) in Greenland.ActaOphthalmol (Copenh). 1988;66:467–8 10.1111/j.1755-3768.1988.tb04042.x 3195326

[7] Rao V, Doctor M, Rao G. Prevalence and Prognosis of Pseudoexfoliation Glaucoma in Western India.Asia Pac J Ophthalmol (Phila). 2015;4:121–7 26065357 10.1097/APO.0b013e3182a0af43

[8] Founti P, Haidich AB, Chatzikyriakidou A, Salonikiou A, Anastasopoulos E, Pappas T, Lambropoulos A et al Ethnicity-Based Differences in the Association of LOXL1 Polymorphisms with Pseudoexfoliation/Pseudoexfoliative Glaucoma: A Meta-Analysis. Ann Hum Genet. 2015;79:431–50 doi: 10.1111/ahg.12128 26404116 10.1111/ahg.12128

[9] Rao A, Padhy D. Pattern of pseudoexfoliation deposits on the lens and their clinical correlation—clinical study and review of literature.PLoSOne. 2014 12 5;9(12):e113329 doi: 10.1371/journal.pone.0113329 25478872 10.1371/journal.pone.0113329 PMC4257528

[10] Anastasopoulos E, Founti P, Topouzis F.Update on pseudoexfoliation syndrome pathogenesis and associations with intraocular pressure, glaucoma and systemic diseases.CurrOpinOphthalmol. 2015;26:82–9 10.1097/ICU.0000000000000132 25594764

[11] Padhy B, Nanda GG, Chowdhury M, Padhi D, Rao A, Alone DP. Role of an extracellular chaperone, Clusterin in the pathogenesis of Pseudoexfoliation Syndrome and Pseudoexfoliation Glaucoma.Exp Eye Res. 2014;127:69–76 doi: 10.1016/j.exer.2014.07.005 25057782 10.1016/j.exer.2014.07.005

[12] Schlötzer-Schrehardt U. New pathogenetic insights into pseudoexfoliation syndrome/glaucoma. Therapeutically relevant?Ophthalmologe. 2012;109:944–51 10.1007/s00347-012-2531-1 23053330

[13] Konstas AG, Tsironi S, Ritch R. Current concepts in the pathogenesis and management of exfoliation syndrome and exfoliative glaucoma. ComprOphthalmol Update. 2006;7:131–41. 16882401

[14] Vesti E, Kivelä T. Exfoliation syndrome and exfoliation glaucoma. ProgRetin Eye Res. 2000;19:345–68 10.1016/s1350-9462(99)00019-1 10749381

[15] Puska PM. Unilateral exfoliation syndrome: conversion to bilateral exfoliation and to glaucoma: a prospective 10-year follow-up study. J Glaucoma. 2002;11:517–24 12483098 10.1097/00061198-200212000-00012

[16] Arnarsson A, Damji KF, Sasaki H, Sverrisson T, Jonasson F. Pseudoexfoliation in the reykjavik eye study: five-year incidence and changes in related ophthalmologic variables.Am J Ophthalmol. 2009;148:291–7 doi: 10.1016/j.ajo.2009.03.021 19427619 10.1016/j.ajo.2009.03.021

[17] Vijaya L, Asokan R, Panday M, Choudhari NS, Sathyamangalam RV, Velumuri L et al The Prevalence of Pseudoexfoliation and the Long-term Changes in Eyes With Pseudoexfoliation in a South Indian Population. J Glaucoma. 2016;25:e596–602. doi: 10.1097/IJG.0000000000000276 25950660 10.1097/IJG.0000000000000276

[18] Vijaya L, Asokan R, Panday M, Choudhari NS, Ve Ramesh S, Velumuri L et al Six-Year Incidence and Baseline Risk Factors for Pseudoexfoliation in a South Indian Population: The Chennai Eye Disease Incidence Study.Ophthalmology. 2015;122:1158–64. doi: 10.1016/j.ophtha.2015.02.007 25795479 10.1016/j.ophtha.2015.02.007

[19] Al-Saleh SA, Al-Dabbagh NM, Al-Shamrani SM, Khan NM, Arfin M, Tariq M et al Prevalence of ocular pseudoexfoliation syndrome and associated complications in Riyadh, Saudi Arabia.Saudi Med J. 2015;36:108–12 doi: 10.15537/smj.2015.1.9121 25630014 10.15537/smj.2015.1.9121 PMC4362197

[20] Mitchell P, Wang JJ, Hourihan F.The relationship between glaucoma and pseudoexfoliation: the Blue Mountains Eye Study.Arch Ophthalmol. 1999;117:1319–24. 10532440 10.1001/archopht.117.10.1319

[21] Anastasopoulos E, Topouzis F, Wilson MR et al Characteristics of pseudoexfoliation in the Thessaloniki Eye Study.J Glaucoma. 2011;20:160–6 doi: 10.1097/IJG.0b013e3181d9d8bd 20436360 10.1097/IJG.0b013e3181d9d8bd

[22] Olawoye OO, Pasquale LR, Ritch R. Exfoliation syndrome in sub-Saharan Africa.IntOphthalmol. 2014;34:1165–73. 10.1007/s10792-014-9953-5 24844849

[23] Ren R, Ding J, Wang N, Teng CC, de Moraes GV, Jonas JB et al Clinical Signs and Characteristics of Exfoliation Syndrome and Exfoliative Glaucoma in Northern China. Asia Pac J Ophthalmol (Phila). 2015;4:86–8:26065350 10.1097/APO.0000000000000106

[24] Sood NN.Prevalence of pseudoexfoliation of the lens capsule in India.ActaOphthalmol (Copenh). 1968;46:211–4. 10.1111/j.1755-3768.1968.tb05179.x 5755679

[25] Landers J, Henderson T, Craig J. Prevalence of pseudoexfoliation syndrome in indigenous Australians within central Australia: The Central Australian Ocular Health Study. ClinExpOphthalmol. 2012;40:454–7. 10.1111/j.1442-9071.2011.02696.x 21902787

[26] Thessaloniki eye study: the importance of recognizing pseudoexfoliation.Parrish RK 2nd.Am J Ophthalmol. 2009;148:482–3. doi: 10.1016/j.ajo.2009.07.005 19782795 10.1016/j.ajo.2009.07.005

[27] Miglior S, Bertuzzi F.Exfoliative glaucoma: new evidence in the pathogenesis and treatment.Prog Brain Res. 2015;221:233–41 doi: 10.1016/bs.pbr.2015.06.007 26518081 10.1016/bs.pbr.2015.06.007

[28] Anastasopoulos E, Founti P, Topouzis F. Update on pseudoexfoliation syndrome pathogenesis and associations with intraocular pressure, glaucoma and systemic diseases. CurrOpinOphthalmol. 2015;26:82–9 10.1097/ICU.0000000000000132 25594764

[29] Vazquez LE, Lee RK. Genomic and proteomic pathophysiology of pseudoexfoliation glaucoma.IntOphthalmolClin. 2014;54:1–13. 10.1097/IIO.0000000000000047 PMC4182319 25171640

[30] Henke V, Naumann GO. Incidence of the pseudo-exfoliation syndrome in enucleated eyes. KlinMonblAugenheilkd. 1987;190:173–5. 10.1055/s-2008-1050350 2953933

