# Supplementary material for: Clinical spectrum of pseudoexfoliation syndrome—An electronic records audit
Source: PLoS One. 2017 Oct 27;12(10):e0185373. doi: 10.1371/journal.pone.0185373 (PMC5659605; doi:10.1371/journal.pone.0185373)
Supplement: S1 Table — (PDF) [file pone.0185373.s001.pdf]

Table S1: Visual acuity in eyes with pseudoexfoliation syndrome, ocular hypertension and exfoliation glaucoma.

Snellen Visual acuity	Pseudoexfoliation Syndrome<br>N=1693	Pseudoexfoliation with OHT<br>N= 234	Pseudoexfoliation Glaucoma<br>N= 447
20/20-20/40 (n)<br>%	309<br>18.2%	50<br>21.3%	82<br>18.4%
20/40-20/200 (n)<br>%	587<br>34.4%	103<br>44.1%	128<br>28.6%
20/200-20/800 (n)<br>%	654<br>38.6%	43<br>18.3%	169<br>37.8%
<20/800 (n)<br>%	143<br>8.8%	38<br>16.3%	68<br>15.2%
